# ﻿The new genus *Purpurata* (Lepidoptera, Crambidae, Spilomelinae), with descriptions of two new species from China

**DOI:** 10.3897/zookeys.1219.131102

**Published:** 2024-12-02

**Authors:** Xin-Lei Xue, Xiao-Qiang Lu, Xi-Cui Du

**Affiliations:** 1 College of Plant Protection, Southwest University, Chongqing 400715, China Southwest University Chongqing China; 2 Yibin Academy of Southwest University, Yibin, Sichuan 644000, China Yibin Academy of Southwest University Yibin China; 3 Guangyuan Center for Disease Control and Prevention, Guangyuan, Sichuan 628040, China Guangyuan Center for Disease Control and Prevention Guangyuan China

**Keywords:** DNA barcode, identification key, morphology, moths, taxonomy

## Abstract

The male genitalia characters of four species, *Botysiopasalis* Walker, 1859, *Pleuroptyaobfuscalis* Yamanaka, 1998, *Botysplagiatalis* Walker, 1859 and *Pataniashompen* Singh et Ahmad, 2022, placed in the genus *Patania* Moore, 1888 before the present study, do not conform to the diagnosis of *Patania*. A new genus, *Purpurata***gen. nov.**, is established for these four species, and two new species, *Purpuratadirecta***sp. nov.** and *Purpuratalurida***sp. nov.** are described based on their external morphology and genitalia characters. *Purpuratadirecta***sp. nov.** is designated as the type species of the new genus. Five species of the new genus were clearly separated from *Patania* species in the Maximum likelihood phylogenetic tree constructed based on COI sequence data. Compared to *Patania*, the new genus *Purpurata* exhibits distinctive characters in male genitalia: the uncus is short, broad, and arc-shaped posteriorly; the gnathos is present and setose, or reduced; and the fibula is very small and setose. In addition, *Pataniaclava* (Xu & Du), **syn. nov.** is synonymized with *Purpurataiopasalis***comb. nov.** An identification key to species of the new genus is presented based on morphological characters of habitus and genitalia. Images of the habitus and genitalia are provided.

## ﻿Introduction

The genus *Patania* Moore, 1888 was assigned to the Spilomelinae tribe Agroterini Acloque, 1897 by Mally et al. (2019). Many species of *Patania* were previously recorded in *Pleuroptya* Meyrick, 1890 (Munroe 1983, 1995; Shaffer et al. 1996; Leraut 2005; Du 2009; Kirpichnikova 2009; Heppner 2012; Sasaki and Yamanaka 2013), which was synonymized with *Patania* by Kirti and Gill (2007). During our study of the genus *Patania*, we found that four species, *P.iopasalis* (Walker, 1859), *P.obfuscalis* (Yamanaka, 1998), *P.plagiatalis* (Walker, 1859) and *P.shompen* Singh et Ahmad, 2022, exhibited some distinctive characters in the genitalia, such as on the uncus, gnathos, fibula, and apophyses anteriores, which did not correspond to the diagnostic characters of *Patania*. Additionally, some genitalia characters of these four species, such as the fibula, cornutus, and corpus bursae, did not correspond to the characters of another similar genus, i.e., *Nagiella* Munroe, 1976. Therefore, after systematically comparing the morphological characters of these four species with *Patania* and *Nagiella* species, and based on the phylogenetic analysis of DNA barcode data, we establish a new genus, *Purpurata* gen. nov., for these four known species and describe two new species.

## ﻿Materials and methods

### ﻿Taxon sampling

Specimens were collected by light trap at night and killed by ammonium hydroxide or ethyl acetate. The examined pinned specimens, including all type specimens of the new species, are deposited in the Insect Collection, Southwest University, Chongqing, China (**SWU**), except for six specimens of two known species, which are deposited in the Insect Collection of Nankai University (**NKU**). The corresponding author examined comparative specimens of *Botysiopasalis* and some *Patania* species, which were deposited in the Natural History Museum, London, United Kingdom (**NHMUK**). We extracted and obtained 11 sequences from five species of the new genus *Purpurata*, seven sequences from three *Patania* species, and five sequences from two *Nagiella* species (Table 1). All these sequences have been uploaded to NCBI. In addition, four sequences of two *Agrotera* species, one sequence of one *Patania* species, two sequences of one *Pycnarmon* species and six sequences of two *Nosophora* species were downloaded from NCBI and BOLD. Among them, *Agrotera* is the type genus of Agroterini (Mally et al. 2019), *Patania* and *Nagiella* are similar genera to *Purpurata*, and *Pycnarmanpantherata* (Butler, 1878) and *Nosophora* are closely related to *Botysiopasalis* (Matsui et al. 2022), one of the focal taxa in the present study. We included the type species of *Patania*, *Botysconcatenalis* Walker, 1866, in the analysis to deduce the monophyly of the new genus *Purpurata*. Therefore, the five genera *Patania*, *Nagiella*, *Agrotera*, *Pycnarmon*, and *Nosophora* are used as outgroups in this study. The information on specimens used for mitochondrial COI gene sequencing and phylogenetic analysis is provided in Table 1.

**Table 1.** Sample information for species of *Purpurata* gen. nov. and the outgroups.

Species	Sequence ID	Location (China, except for last four)	NCBI and BOLD accession no.
*Purpurataiopasalis* comb. nov.	XD1401067	Wuzhishan, Hainan	PQ463661
	LXQ180076	Diaoluoshan, Hainan	PQ463660
	XD1401014	Diaoluoshan, Hainan	PQ463662
*Purpuratadirecta* sp. nov.	XD1401032	Wuzhishan, Hainan	PQ463664
	XD1401110	Diaoluoshan, Hainan	PQ463663
*Purpurataplagiatalis* comb. nov.	XD1500022	Mulun, Guangxi	PP865067
*Purpurataobfuscalis* comb. nov.	LXQ180070	Haugaoxi, Sichuan	PP865068
	LXQ180071	Haugaoxi, Sichuan	PP865069
	XD1405336	Huagaoxi, Sichuan	KU143853
*Purpuratalurida* sp. nov.	XD1401079	Diaoluoshan, Hainan	PP865070
	LXQ180262	Changyang, Hubei	PP865071
*Pataniabalteata*	XD1405399	Haugaoxi, Sichuan	KU143838
	XD1405300	Haugaoxi, Sichuan	KU143837
	XD1405441	Haugaoxi, Sichuan	KU143839
*Pataniachlorophanta*	XD1404265	Jinzhongshan, Guangxi	KU058652
	XD1404239	Jinzhongshan, Guangxi	KU058653
	XD1401035	Wuzhishan, Hainan	KU058654
*Pataniaconcatenalis*	XD1401058	Diaoluoshan, Hainan	KU143840
	Pyr000116	Lingshui, Hainan	CNPYA116-10
*Pycnarmonpantherata*	Pyr000126	Yuanqu, Shanxi	CNPYA126-10
	Pyr000127	Yuanqu, Shanxi	CNPYA127-10
*Nagiellahortulatoides*	LXQ180100	Huanglianshan, Yunnan	PQ463665
	LXQ180099	Huanglianshan, Yunnan	PQ463666
	LXQ180217	Huanglianshan, Yunnan	PQ463667
*Nagiellaquadrimaculalis*	XD1405327	Haugaoxi, Sichuan	KU143854
	XD1402131	Bawangling, Hainan	PP865072
*Nosophoradispilalis*	Pyr001429	Nanning, Guangxi	CNPYD1429-10
	Pyr001430	Nanning, Guangxi	CNPYD1430-10
	Pyr001431	Nanning, Guangxi	CNPYD1431-10
*Nosophorasemitritalis*	Pyr000949	Yuanqu, Shanxi	CNPYD949-10
	Pyr000950	Yuanqu, Shanxi	CNPYD950-10
	Pyr000951	Yichang Wufeng, Hubei	CNPYD951-10
*Agroteranemoralis*	ODOPE217-11	Bavaria, Germany	KX045648
	PHLAC175-10	South Tyrol, Italy	JF859792
*Agroterabasinotata*	ANICN229-10	Queensland, Australia	HQ952613
	ANICN230-10	Queensland, Australia	HQ952614

The preparation of genitalia slides primarily followed Li and Zheng (1996). The habitus images were taken with a digital camera (Canon EOS 5D), and the genitalia images were taken with a digital camera (Leica DFC 450) attached to a stereomicroscope (Leica M205 A).

### ﻿DNA extraction, PCR amplification, sequencing

A total of 15 species, including five species of *Purpurata* gen. nov. and ten outgroup species, were included for PCR analysis and DNA sequencing (Table 1). Total DNA was extracted from dried legs using the TIANGEN DNA extraction kit according to the manufacturer’s instructions. The 658-base pair (bp) barcode region of COI was PCR amplified using primers LepF1 and LepR1 (Hajibabaei et al. 2006). After verifying PCR products by running them on a 1% agarose gel, sequencing was conducted by Shanghai Sangon Biotechnology Co., Ltd (Shanghai, China) using the same primers as those used in PCR.

### ﻿Data analysis

All COI sequences were aligned manually using Align-Muscle in MEGA 7.0, and were translated into amino acid sequences for visual correction. Intraspecific and interspecific genetic distances were calculated based on the Kimura 2-parameter (K2P) distance model (Kimura 1980). Phylogenetic analysis was performed based on a phylogenetic tree constructed using the Maximum likelihood (ML) method with 1,000 bootstrap replications (Saitou and Nei 1987), in which three *Patania* species, two *Nagiella* species, one *Pycnarmon* species, two *Nosophora* species and two *Agrotera* species were chosen as the outgroups.

## ﻿Results

### ﻿DNA sequence analysis

A total of 11 COI sequences from five species of *Purpurata* gen. nov. were obtained. In total, 36 COI sequences from *Purpurata* species and the outgroup species were analyzed. The dataset contained no obvious pseudogenes, indicating the correct target gene sequence was amplified and sequenced.

**Figure 1.**
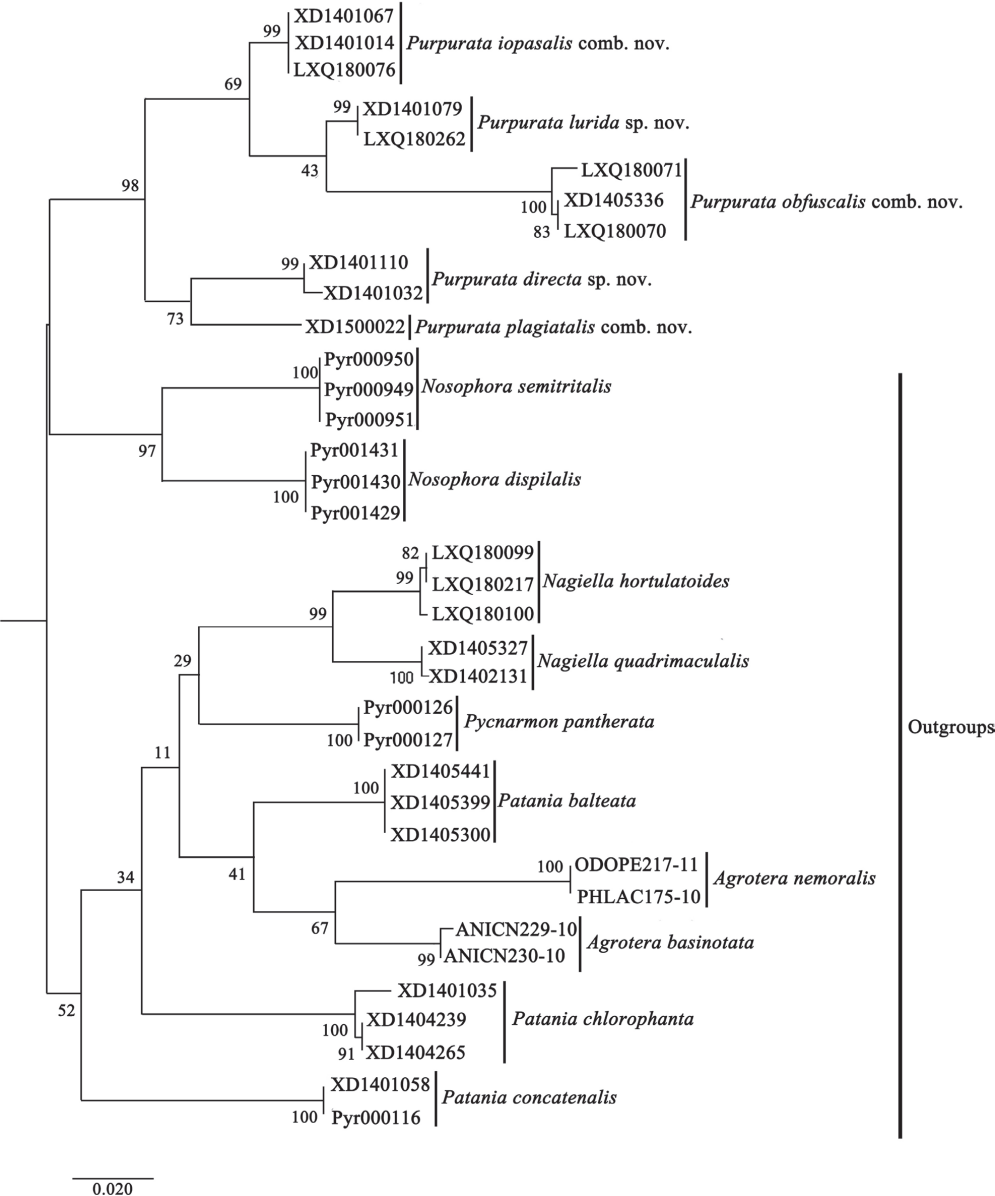
Phylogenetic hypothesis of relationships among five species of *Purpurata* gen. nov. and ten species of five outgroup genera inferred from a Maximum likelihood (ML) analysis of the DNA barcode data. Numbers near the branches are bootstrap support values based on 1000 replicates.

The ML phylogenetic tree shows five monophyletic branches for *Purpurata* gen. nov., corresponding to the five morphological species *P.iopasalis* comb. nov., *P.lurida* sp. nov., *P.obfuscalis* comb. nov., *P.directa* sp. nov., and *P.plagiatalis* comb. nov. (Fig. 1). These branches form a well-supported (98% Bootstrap support) monophylum that is sister to *Nosophora*. *Patania*, with three sampled species, including the type species *P.concatenalis*, is polyphyletic, with *Nagiella*, *Pycnarmon*, and *Agrotera* nested within. The intraspecific genetic distances within *Purpurata* range from 0.00% to 0.01%, and interspecific genetic distances range from 3.58% to 9.49% (Table 2). The interspecific genetic distances between the ingroup species and outgroup species range from 9.15% to 13.54% (Table 2).

**Table 2.** Kimura 2-parameter genetic distances (as percent) calculated within (in bold) and between *Purpurata* species and the outgroups.

	1	2	3	4	5	6	7	8	9	10	11	12	13	14	15
1. *Purpurataiopasalis* comb. nov.	**0.00**														
2. *Purpurataobfuscalis* comb. nov.	8.72	**0.01**													
3. *Purpuratadirecta* sp. nov.	6.93	9.49	**0.00**												
4. *Purpurataplagiatalis* comb. nov.	6.84	8.36	5.45	**0.00**											
5. *Purpuratalurida* sp. nov.	3.58	6.52	8.28	7.85	**0.00**										
6. *Pataniaconcatenalis*	9.91	12.76	10.90	10.80	11.87	**0.00**									
7. *Pataniabalteata*	10.59	13.09	10.25	9.38	12.36	10.60	**0.00**								
8. *Pataniachlorophanta*	9.15	12.50	11.36	11.06	12.07	12.07	8.53	**0.00**							
9. *Nosophoradispilalis*	10.95	11.72	11.40	10.94	11.31	10.25	12.01	10.53	**0.00**						
10. *Nosophorasemitritalis*	10.77	11.14	10.34	10.07	10.25	11.51	12.36	11.41	7.20	**0.00**					
11. *Pycnarmonpantherata*	10.42	12.23	11.21	10.95	11.30	10.77	8.87	9.39	10.07	11.84	**0.00**				
12. *Nagiellahortulatoides*	11.30	11.43	12.47	10.43	12.85	10.90	9.21	10.95	10.89	11.84	9.40	**0.00**			
13. *Nagiellaquadrimaculalis*	11.39	12.29	11.58	10.68	12.64	10.69	8.95	10.98	10.33	11.58	8.96	4.47	**0.00**		
14. *Agroteranemoralis*	11.92	13.54	12.29	11.21	13.18	10.86	9.81	11.98	11.56	11.03	11.21	11.03	11.65	**0.00**	
15. *Agroterabasinotata*	10.24	12.92	10.43	10.25	10.94	9.74	7.18	11.15	11.93	10.96	9.22	10.43	9.46	8.37	**0.00**

### ﻿Taxonomic account

#### ﻿ Purpurata gen. nov.

Taxon classificationAnimaliaLepidopteraCrambidae

57F95D25-A1F2-5BEB-AFC2-378B430683E5

https://zoobank.org/B30E1964-1BD5-47CD-9196-BA496B0FA390

##### Type species.

*Purpuratadirecta* sp. nov., here designated.

##### Diagnosis.

**Figures 2–5.**
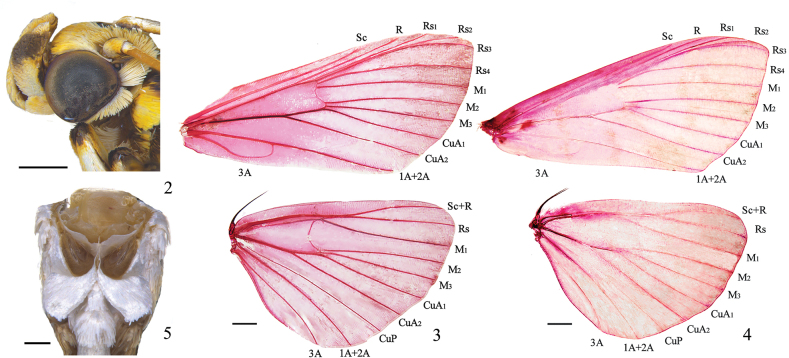
*Purpuratadirecta* sp. nov. **2** head ♂ **3** wing venation ♂ **4** wing venation ♀ **5** tympanal organ. Scale bars: 0.5 mm (**2, 5**); 1.0 mm (**3, 4**).

*Purpurata* is readily distinguished from its phylogenetic sister genus *Nosophora* (type species: *Botysdispilalis* Hampson, 1896) by the yellow wings with their purple-brown lines and patches, while in *Nosophora*, the wings are dark shiny brown and feature a large, comma-shaped spot in the forewing. Furthermore, the vertex of the head is hollowed out in males of *N.dispilalis* (see Lederer 1863: pl. 4 fig. 24 (misidentified as *N.chironalis*); Hampson 1896: 288), whereas it is rounded in *Purpurata* species (Fig. 2). In *Purpurata*, compared to the similar genera *Patania* and *Nagiella*, the uncus is arc-shaped on the posterior margin, the valva is shorter and broader than those of the latter two, and the fibula is shorter and smaller in male genitalia; the apophyses anteriores is not broadened or very slightly broadened near the base in the female genitalia. Comparatively, in *Patania* and *Nagiella*, the uncus is generally trapezoidal (except that *N.bispina* has a rather short and broad uncus, with rounded posterior margin), and the fibula are generally well developed and sclerotized; the apophyses anteriores broaden rhomboidally near the base. In addition, in *Purpurata*, the gnathos is present and setose apically, generally undeveloped (except that *P.iopasalis* and *P.shompen* have well-developed gnathos). *Patania* species usually lack a gnathos, and a few species have a gnathos but no setae at the apex. *Nagiella* species generally have a gnathos, but no setae at the apex. In *Purpurata*, the phallus has a protruding sclerotized structure at the posterior end, with a thick needle-like or spine-like cornutus and a brush-like cornutus. Comparatively, the phallus of *Patania* and *Nagiella* lack a protruding sclerotized structure posteriorly. *Patania* species have, or lack, a cornutus (if present, diverse morphologically), and *Nagiella* species generally lack a cornutus (except for *N.bispina* with a curved hook cornutus). The compared characters here are present in the type species of *Patania* and *Nagiella*, *Botysconcatenalis*, and *Nagiadesmialis* respectively, which were fully investigated in our study.

##### Description.

***Habitus*.** Body and wings yellow, with purple-brown wing markings. Frons rounded. Labial palpus upturned, exposed 3^rd^ joint short and blunt (Fig. 2). Antenna filiform, male with ventral cilia. Forewing with length of cell ~ 1/2 of wing; discocellulars incurved; R from cell at ~ 5/6 above; Rs_2_ anastomosed with Rs_3_ ~ 3/5 of Rs_3_ beyond cell; Rs_1_ close to Rs_2_+s_3_; Rs_4_ slightly curved towards Rs_2_+s_3_ at base; M_2_, M_3_ and CuA_1_ from posterior angle of cell and uniformly spaced at the base; CuA_2_ from cell at ~ 2/3 below. Hindwing with length of cell ~ 1/3 of wing; discocellulars incurved; Sc+R anastomosed with Rs ~ 1/5 beyond the cell; M_2_, M_3_ and CuA_1_ separately from posterior angle cell; CuA_2_ from cell at 2/3 below (Figs 3, 4). Abdomen yellow dorsally; 1^st^ and 2^nd^ tergites with black spots laterally and 7^th^ tergites black posteriorly in male. Tympanal organs with praecinctorium strongly bifid (Fig. 5).

***Male genitalia*.** Uncus short and broad, with arc-shaped posterior margin, except for *P.iopasalis* with narrowed apex of uncus. Gnathos present and bearing setae apically, or vestigial to a transverse band. Valva ligulate, relatively broad and short generally. Fibula small and membranous, with setae. Sacculus undeveloped. Phallus cylindrical, with a protruding sclerotized structure posteriorly, with a thick needle-like or spine-like cornutus and a brush-like cornutus composed of a spine cluster, and a granular sclerotized area near the middle.

***Female genitalia*.** Apophyses anteriores longer than apophyses posteriores, occasionally slightly broadened near base. Antrum sclerotized. Ductus bursae usually long and slender. Corpus bursae round or oval, usually with a round signum.

##### Distribution.

This genus is mainly distributed in the Palaearctic and Oriental realms. And *P.plagiatalis* is also found in the Neotropical and Australasian realms, *P.iopasalis* is also found in the Australasian realms.

##### Etymology.

The genus name *Purpurata* is derived from the Latin word *purpuratus* meaning purple, indicating the distinctive purple-brown coloration of wing markings. The gender is feminine.

##### Remarks.

This new genus comprises four known species from the genus *Patania* of Spilomelinae, and two new species described in the present study. It corresponds to the main diagnostic characters of Crambidae: forewing with vein R_S4_ free and with oval sclerotization costad at base of vein 1A+2A; bullae tympani open cephalad; tympanum and conjunctivum lying at a blunt angle; praecinctorium present; and male genitalia without uncus arms. Further, it corresponds to the main diagnostic characters of Spilomelinae: fornix tympani projecting ventrad from tympanic frame; praecinctorium bilobed; retinacular hook absent; and females with two frenular bristles. Moreover, based on the Agroterini characters stated in Mally et al. (2019), labial palps upturned and 3^rd^ labial palpomere directed dorsally; uncus with a broad base, head chaetae simple and unsplit; the ratio between saccus length and sacculus breadth > 1, we place this genus in the tribe Agroterini.

### ﻿Key to *Purpurata* species based on morphology of habitus and genitalia

**Table:** 

1	Postmedial line of forewing punctiform between M_2_ and CuA_2_ (Figs 7, 11); phallus posteriorly with a nail head-like, rectangular, or rounded protruding sclerite (Figs 14c, 19c)	**2**
–	Postmedial line of forewing dentate or linear between M_2_ and CuA_2_ (Figs 6, 8–9, 10); phallus posteriorly with a finger-like or oval protruding sclerite (Figs 12c, 15c, 17c)	**4**
2	Gnathos vestigial to a narrow band (Fig. 19a); sacculus with a lamellar projection near distal end (Fig. 19); phallus posteriorly with a rounded protruding sclerite, and a short thorn-like cornutus near end, besides needle-like cornutus and brush-like cornutus (Fig. 19c)	***P.plagiatalis* comb. nov.**
–	Gnathos thick finger-like (Fig. 14a); sacculus without lamellar projection; phallus posteriorly with a nail head-like or rectangular sclerite, and without short thorn-like cornutus near distal end, besides needle-like cornutus and brush-like cornutus (Fig. 14c)	**3**
3	Postmedial line of hindwing punctiform between M_2_ and CuA_2_ (Fig. 7); phallus posteriorly with a nail head-like sclerite (Fig. 14c)	***P.iopasalis* comb. nov.**
–	Postmedial line of hindwing linear between M_2_ and CuA_2_; phallus posteriorly with a rectangular sclerite	***P.shompen* comb. nov.**
4	Postmedial line of forewing linear between M_2_ and CuA_2_ (Figs 8, 9); phallus posteriorly with an oval sclerite (Fig. 15c); gnathos transverse lamina (Fig. 15a)	***P.directa* sp. nov.**
–	Postmedial line of forewing dentate between M_2_ and CuA_2_ (Figs 6, 10); phallus posteriorly with a finger-like sclerite (Figs 12c, 17c); gnathos vestigial to a narrow band (Figs 12a, 17a)	**5**
5	Uncus short and broad (Fig. 17a), triangular; valva with distal 1/3 narrowed very gradually and rounded apically (Fig. 17)	***P.lurida* sp. nov.**
–	Uncus semicircular (Fig. 12a); valva with distal 1/3 narrowed gradually and pointed apically (Fig. 12)	***P.obfuscalis* comb. nov.**

#### ﻿ Purpurata obfuscalis

Taxon classificationAnimaliaLepidopteraCrambidae

(Yamanaka, 1998) comb. nov.

D40F2DDD-07E8-59D1-A011-C98715592E9D

**Figures 6–11.**
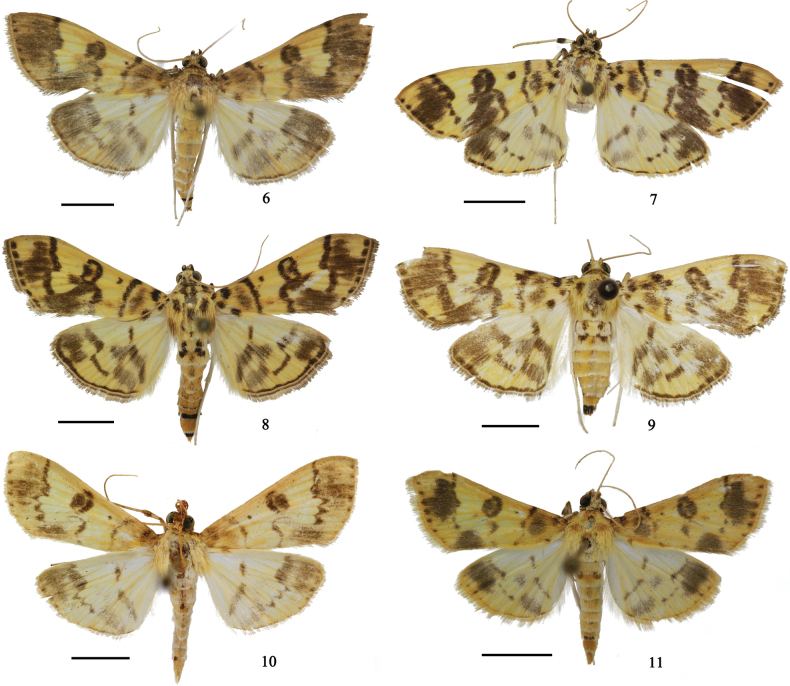
Habitus of *Purpurata* species **6***P.obfuscalis* ♂ **7***P.iopasalis* ♂ **8***P.directa* sp. nov. holotype ♂ **9***P.directa* sp. nov. paratype ♀ **10***P.lurida* sp. nov. holotype ♂ **11***P.plagiatalis* ♂. Scale bars: 0.5 cm.

**Figures 12–16.**
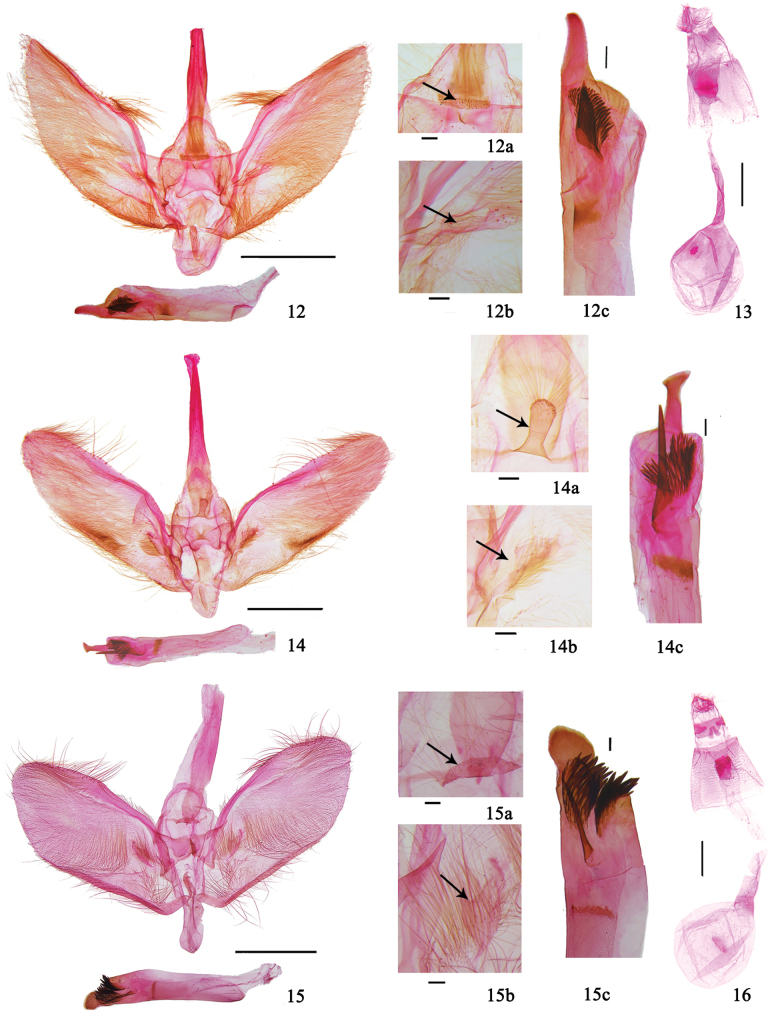
Genitalia of *Purpurata* species **12, 13***P.obfuscalis***12** male, genitalia slide no. XXL23275 **13** female, genitalia slide no. XLJ14221 **14***P.iopasalis*, male, genitalia slide no. XXL23277 **15, 16***P.directa* sp. nov. **15** male, holotype, genitalia slide no. LXQ18315 **16** female, paratype, genitalia slide no. XLJ14147 **12a, 14a, 15a** gnathos **12b, 14b, 15b** fibula **12c, 14c, 15c** cornuti and posterior protrusion of phallus. Scale bars: 1.0 mm (**12–16**); 0.1 mm (**12a–c, 14a–c, 15a–c**).

Figs 6 , 12 , 12a–c , 13

Pleuroptya obfuscalis Yamanaka, 1998: 106. Type locality: Nepal.
Patania obfuscalis : Nuss et al. 2003–2024. Global Information System on Pyraloidea.

##### Material examined.

**China** • **Chongqing Municipality**, 2 ♂♂, 1 ♀, Jinyun Mountain, alt. 550 m, 29 July 2010, Xi-Cui Du & Chao-Wei Bi leg., genitalia slide no.: XLJ14221 ♀ • 1 ♂, Simian Mountain, alt. 1120 m, 17 July 2010, Xi-Cui Du & Li-Fang Song leg. • 2 ♂♂, JinYin Mountain, Qianjiang District, alt. 1100 m, 25–26 July 2012, Jun Zhang & Li-Jun Xu leg. • 1 ♂, Small South Sea, Qianjiang District, alt. 370 m, 21 July 2012, Jun Zhang & Li-Jun Xu leg. • **Sichuan Prov.**, 4 ♂♂, Huagaoxi Nature Reserve, Guandou Village, alt. 763 m, 30 August 2014, Dan-Xu & Xue-Li Wei leg., genitalia slide no.: XDJ15048 ♂ • **Gansu Prov.**, 2 ♂♂, Bifeng Valley, Wen County, alt. 860 m, 2005. 9–10 July 2012, Hai-Li Yu leg. (NKU) • **Yunnan Prov.**, 2 ♂♂, Pianma Village, Lushui County, Nujiang Prefecture, alt. 1889 m, 18 August 2015, Xue-Li Wei leg., genitalia slide no.: LXQ18279 • 2 ♂♂, Cangyuan County, Lincang City, alt. 1242 m, 25 July 2015, Xue-Li Wei leg. • 1 ♂, Jinuo Township, Xishuangbanna, alt. 1100 m, 15 May 2018, Xi-Cui Du leg. • 2 ♂♂, Xishuangbanna Tropical Botanical Garden, alt. 659 m, 28 May 2015, Man-Fei Tao leg. • 2 ♂♂, Daxichang Village, Malipo County, alt. 1465 m, 5 June 2015, Man-Fei Tao leg. • 2 ♂♂, Huanglian Mountain, Honghe Prefecture, alt. 900 m, 23 May 2018, Xi-Cui Du & Xiao Qiang-Lu leg. • **Xizang Autonomous Region**, 1 ♂, Zhangmu Town, alt. 2300 m, 5 August 2017, Jian-Yue Qiu & Hao Xu leg., genitalia slide no.: XXL23275 ♂ • **Guangxi Zhuang Autonomous Region**, 1 ♂, Nonggang National Nature Reserve, Longzhou County, alt. 188 m, 3 August 2011, GuiQing-He leg. • **Guizhou Prov.**, 2 ♂♂, 3 ♀♀, Baishao, Kuankuoshui, alt. 800 m, 10–11, 13 August 2010, Xi-Cui Du leg. • 1 ♂, WengAng village, LiBo County, alt. 1345 m, 20 July 2015, Ji-Ping Wan leg. • **Hainan Prov.**, 4 ♀♀, Wuzhi Mountain, alt.795 m, 18 May 2014, Li-Jun Xu & Dan Xu leg. • 1♂, Diaoluo Mountain, alt. 500 m, 23 May 2014, Li-Jun Xu & Dan Xu leg. • **Shaanxi Prov.**, 1 ♂, Ningshan County, Xunyangba Town, alt. 1400 m, 3 August 2014, Hai-Li Yu leg. • **Hubei Prov.**, 1♂, Pingbaying National Forest Park, Xianfeng County, alt. 280 m, 21 July 1999, Hou-Hun Li leg. (NKU) • 5 ♂♂, 2 ♀♀, Hejiaping Town, Changyang County, alt. 800 m, 18 June 2018, Xi-Cui Du & Xiao Qiang-Lu leg., genitalia slide no.: LXQ18140 ♀ • **Anhui Prov.**, 1 ♂, Tang Kou Town, Huangshan City, 4 August 2004, Jia-Sheng Xu & Jia-Liang Zhang leg. (NKU) • **Zhejiang Prov.**, 1 ♂, Longtang Mountain, alt. 500 m, 22 May 2012, Xiao-Bing Fu leg. • 2 ♂♂, Tianmu Mountain, alt. 400 m, 24–25 July 2011, Xi-Cui Du & Xiao Bing-Fu leg. • **Hong Kong Special Administrative Region**, 1 ♂, Kadoorie Farm, alt. 21 m, 13 April 2007, Hou-Hun Li leg. (NKU).

##### Diagnosis.

This species is distinguished by wings with postmedial line dentate and excurved between M_2_ and CuA_2_ (Fig. 6); gnathos vestigial to a narrow band (Fig. 12a); valva with distal 1/3 narrowed gradually and pointed apically, costa arched medially and bearing a cluster of long setae (Fig. 12); fibula a triangular lamina, with setae medially (Fig. 12b); phallus posteriorly with a finger-like protruding sclerite, a thick, spine-like cornutus and a brush-like cornutus (Fig. 12c).

##### Redescription.

***Habitus* (Fig. 6).** Forewing length 8.0–15.0 mm, wingspan 25.0–33.0 mm. Frons and vertex yellowish brown. Labial palpus with 1^st^ segment yellowish white ventrally, the remainder yellowish brown. Maxillary palpus brown. Antenna yellowish brown, with ventral cilia ~ 1/3 in length of diameter of flagellomere in male. Patagium and tegula yellow, with brown patches. Thorax yellow dorsally, white ventrally. Legs yellowish white, distal end of front tibia black. Wings yellow, with purple-brown lines and patches. Forewing with three small spots at base, another spot near basal dorsum; antemedial line slightly wavy, accompanied by a large elliptical pale patch inside; orbicular stigma a dark brown dot; discoidal stigma reniform, yellow centrally; postmedial line obliquely inward from costa, dentate and excurved between M_2_ and CuA_2_, then incurved to discoidal stigma below and sinuous to inner margin; an irregular large patch between anterior postmedial line and terminal margin, another irregular large patch below discoidal stigma and extended to tornus; a line of small spots along terminal margin. Hindwing with discoidal stigma a short oblique stripe; postmedial line same as forewing before CuA_2_, not apparent afterwards; an irregular large patch near apex beyond anterior postmedial line; a misty band below discoidal stigma, accompanied by another irregular misty wide band extended to tornus. Abdomen yellow dorsally; 1^st^ and 2^nd^ tergites with pale black spots laterally; 7^th^ tergites black posteriorly in male; terminally black in some female individuals.

***Male genitalia*** (Fig. 12). Uncus semicircular. Gnathos vestigial to a narrow band (Fig. 12a). Valva with distal 1/3 narrowed gradually, and pointed apically; costa arched medially and bearing a cluster of long setae. Fibula a triangular lamina, with setae medially (Fig. 12b). Saccus oval. Juxta a broad plate, invaginated anteriorly. Phallus posteriorly with a finger-like protruding sclerite, with a thick spine-like cornutus and a brush-like cornutus composed of a spine cluster (Fig. 12c).

***Female genitalia*** (Fig. 13). Apophyses anteriores ~ 2 × length of apophyses posteriores. Ostium bursae relatively large, antrum broad. Ductus bursae ~ 2 × length of corpus bursae. Corpus bursae nearly rounded, with a round signum.

##### Distribution.

China (Chongqing, Sichuan, Gansu, Xizang, Yunnan, Guangxi, Guizhou, Hainan, Shanaxi, Hubei, Anhui, Zhejiang, Xianggang), Nepal (Yamanaka 1998).

#### ﻿ Purpurata iopasalis

Taxon classificationAnimaliaLepidopteraCrambidae

(Walker, 1859) comb. nov.

DF816B29-8FDC-5A29-84F7-95591BC5F373

Figs 7 , 14 , 14a–c

Botys iopasalis Walker, 1859: 652. Type locality: India (Hindustan). Type depository: NHMUK.
Boty boteralis Walker, 1859: 716. Type locality: Malaysia (Sarawak).
Sylepta [sic] *marcidalis* Swinhoe, 1906: 382.
Pleuroptya iopasalis : Inoue 1982: 1: 343, 2: 234, 454.
Patania clava Xu & Du, 2016: 130, figs 1–4, 9–10. Syn. nov. Type locality: China (Hainan).

##### Material examined.

***Holotype*** of *Pataniaclava* • 1 ♂, **China, Hainan Prov.**, Diaoluo Mountain, alt. 900 m, 23 May 2014, Li-Jun Xu & Dan Xu leg., genitalia slide number XD15056. ***Paratype*** of *P.clava* • 1 ♀, same data as holotype, genitalia slide number XD15050.

##### Additional material.

**China** • **Hainan Prov.**, 1 ♂, Wuzhi Mountain, alt. 795 m, 20 May 2014, Li-Jun Xu & Dan Xu leg., genitalia slide no.: XXL23277.

##### Diagnosis.

This species is similar to *P.obfuscalis* in appearance, but can be distinguished by the larger size (forewing length 13.0–19.0 mm, wingspan 33.0–35.0 mm), antenna with ventral cilia approximately as long as flagellomere diameter in male, postmedial line of fore and hind wings punctiform between M_2_ and CuA_2_ (Fig. 7); uncus nearly triangular (Fig. 14), gnathos thick, finger-like (Fig. 14a), costa of valva without a cluster of setae medially, fibula densely covered with setae (Fig. 14b), phallus posteriorly with a nail head-like protruding sclerite (Fig. 14c) in male genitalia (Fig. 14); ductus bursae ~ 4 × length of corpus bursae, signum absent in female genitalia. In *P.obfuscalis*, the forewing length is 8.0–15.0 mm and wingspan is 25.0–33.0 mm, the ventral cilia of antenna is ~ 1/3 length of flagellomere diameter in male, the postmedial lines of the fore and hind wings are dentate between M_2_ and CuA_2_ (Fig. 6). In male genitalia, the uncus of *P.obfuscalis* is semicircular (Fig. 12), the gnathos is reduced to a narrow band (Fig. 12a), the costa of valva bears a cluster of long setae medially (Fig. 12), the fibula bears setae medially (Fig. 12b), the phallus posteriorly has a finger-like protruding sclerite (Fig. 12c); in female genitalia, the ductus bursae is ~ 2 × as long as the corpus bursae and a rounded signum is present (Fig. 13).

##### Distribution.

China (Yunnan, Hainan, Fujian, Guangdong, Taiwan) (Lu and Guan 1953; Xu and Du 2016), Japan, Indonesia, India, Myanmar, Sri Lanka, Malaysia, Thailand, Timor Leste, Philippines, Caroline Islands, Papua New Guinea, Pakistan, Australia (Hampson 1896; Shibuya 1928, 1929; Klima 1939; Inoue 1982; Wang and Speidel 2000).

##### Remarks.

The uncus and fibula of this species are narrower than in other species of this genus. We found that the external morphology and male genitalia characters of *Pataniaclava* were the same as those of *Purpurataiopasalis*. Their distinctive characters were that the postmedial lines of the fore and hind wings were punctiform between M_2_ and CuA_2_, the gnathos was thick finger-like and setose apically (Walker 1859; Inoue 1982, Yamanaka 1998; Xu and Du 2016). Therefore, *Pataniaclava* is synonymized with *Botysiopasalis*. For a detailed description of this species see Walker (1859) and Xu and Du (2016). Moreover, Xu and Du (2016) described that the labial palpus of *Pataniaclava* was pale yellow with a brown patch near the base, while we observed that this species had a subapical brown spot beside this basal spot, and the subapical brown spot was obscured in some individuals.

#### ﻿ Purpurata directa sp. nov.

Taxon classificationAnimaliaLepidopteraCrambidae

DCBC6FA6-131B-5DB6-9B62-E8E56D69D3FB

https://zoobank.org/2C6FEFF3-EB2B-466D-B087-81CBBB2D6E51

Figs 2 , 8 , 9 , 15 , 15a–c , 16

Patania iopasalis : Xu and Du 2016: 132, figs 5–8. Type locality: India (misidentification).

##### Diagnosis.

This species is very similar to *P.iopasalis* in appearance, but can be distinguished by ventral cilia ~ 1/3 length of flagellomere diameter in male, antemedial line of forewing relatively straight and slightly inclined outward, postmedial line of fore and hind wings smooth linear between M_2_ and CuA_2_ (Figs 8, 9); uncus semicircular, gnathos a transverse lamina, and valva broader than the latter in male genitalia (Fig. 15); a signum present in female genitalia (Fig. 16). In *P.iopasalis*, the ventral cilia are approximately equal in length of flagellomere diameter in male, the antemedial line is slightly wavy, the postmedial line of fore and hind wings punctiform between M_2_ and CuA_2_ (Fig. 7); the uncus is nearly triangular, and the gnathos is finger-like in the male genitalia (Fig. 14a); and the signum is absent in the female genitalia.

##### Type material.

***Holotype***: ♂ pinned, with genitalia in a separate slide. **China** • **Hunan Prov.**, Huping Mountain, Shimen County, alt. 350 m, 6 June 2017, Jian-Yue Qiu & Hao Xu leg., genitalia slide number: LXQ18315. ***Paratypes***: pinned, some with genitalia in the separate slides, respectively. **China** • 6 ♂♂, same data as holotype • **Hubei Prov.**, 1 ♂, Hejiaping Town, Changyang County, alt. 800 m, 18 May 2018, Xian-Qiang Lu & Xi-Cui Du leg. • **Chongqing Municipality**, 2 ♀♀, Daheba, Jinfo Mountain, alt. 600 m, 14, 17 July 2017, Shi-Man Bu leg • **Sichuan Prov.**, 2 ♂♂, Emei Mountain, alt. 863 m, 17 July 2011, Jian-Bo Cao leg. • **Guizhou Prov.**, 3 ♂♂, Baishao, Kuankuoshui, alt. 800 m, 10 August 2010, Xi-Cui Du leg., genitalia slide number: HGQ13018, HGQ13019 • 3 ♂♂, 1 ♀, Wengang village, Libo County, alt. 1345 m, 20 July 2015, Ji-Ping Wan leg. • **Yunnan Prov.**, 1 ♂, Baihualing Village, Baoshan City, alt. 1520 m, 13 July 2007, Dan-Dan Zhang leg. • 1 ♂, Daxichang Village, Malipo County, alt. 1465 m, 7 August 2007, Man-Fei Tao leg. • **Hainan Prov.**, 1 ♂, Bawangling National Forest Park, 11 June 2010, Li Kang leg., genitalia slide number: XLJ14105 • 3 ♂♂, 1 ♀, Wuzhi Mountain, alt. 795 m, 18, 19, 21 May 2014, Li-Jun Xu & Dan Xu leg., genitalia slide number: XD15024 ♂, XLJ14146 ♂, XLJ14147 ♀ • 1 ♂, Diaoluo Mountain, alt. 500 m, 25 May 2014, Li-Jun Xu & Dan Xu leg., genitalia slide number: XD15049 • **Guangxi Zhuang Autonomous Region**, 3 ♂♂, Nonggang National Nature Reserve, Longzhou County, alt. 188 m, 25, 27, 30 July 2011, Gui-Qing He leg., genitalia slide number: XLJ14083 (being identified as *Pataniaiopasalis* by Xu and Du (2016)), XLJ13199 • 3 ♂♂, Nonggang National Nature Reserve, Longzhou County, alt. 188 m, 2 August 2011, Gui-Qing He leg., genitalia slide number: XLJ13164 • 5 ♂♂, Mulun Nature Reserve, alt. 288 m, 21 July 2015, Dan Xu leg. • **Guangdong Prov.**, 5 ♂♂, Renhua County, Danxia Mountain, alt. 408 m, 31 May 2018, Feng-Xia He leg.

##### Description.

***Habitus*** (Figs 2, 8, 9). Forewing length 9.0–13.5 mm, wingspan 21.0–30.0 mm. Frons and vertex yellow. Labial palpus yellowish white, with distal part of 2^nd^ segment brown. Maxillary palpus yellowish white basally, brown near distal end. Antenna yellowish brown, with brown spots on scape, ventral cilia ~ 1/3 in length of diameter of flagellomere in male. Patagium and tegula yellow, with brown patches. Thorax yellowish brown dorsally, white ventrally. Legs pale yellow, front coxa and middle tibia with outer sides black at the base and distal end, front tibia with distal end black. Wings yellow, with purple-brown lines and patches. Forewing with three small spots at base, another spot near basal dorsum; antemedial line relatively straight, slightly inclined outward, accompanied by a large elliptical pale patch inside; orbicular stigma a dark brown dot; discoidal stigma reniform, yellow centrally, connected to postmedial line posteriorly; postmedial line slightly obliquely inward from costa, straightly excurved between M_2_ and CuA_2_, then incurved to discoidal stigma below and sinuous to inner margin; an irregular large patch between anterior postmedial line and terminal margin, another irregular large patch below discoidal stigma and extended to tornus; a line of small spots along terminal margin. Hindwing with discoidal stigma a short oblique stripe; postmedial line same as forewing before CuA_2_, not apparent afterwards; an irregular large patch near apex beyond anterior postmedial line; a band below discoidal stigma slightly inclined towards tornus, accompanied by another irregular misty wide band extended to tornus. Cilia of fore and hind wings purple-brown, white basally. Abdomen yellow dorsally, white ventrally; 1^st^ and 2^nd^ tergites with black spots laterally; 7^th^ and 8^th^ tergites black posteriorly in male; terminally black in female.

***Male genitalia*** (Fig. 15). Uncus semicircular. Gnathos a transverse lamina, with posterior end arc-shaped and setose (Fig. 15a). Valva broad tongue-shaped, with long setae along distal costa; sacculus a narrowed band; fibula a triangular lamina, setose basally (Fig. 15b). Saccus cylindrical, with rounded end. Juxta a narrowed plate. Phallus posteriorly with an oval protruding sclerite, with a thick, needle-like cornutus and a brush-like cornutus composed of a spine cluster (Fig. 15c).

***Female genitalia*** (Fig. 16). Apophyses anteriores ~ 2 × length of apophyses posteriores. Ductus seminalis originating from antrum. Ductus bursae ~ 2 × length of corpus bursae. Corpus bursae nearly rounded, with a short transverse bar-like signum.

##### Distribution.

China (Chongqing, Sichuan, Guizhou, Yunnan, Hainan, Hubei, Hunan, Guangdong, Guangxi).

##### Etymology.

The species name *directa* is derived from the Latin word *directus*, an adjective, meaning straight, indicating the antemedial line of forewing relatively straight.

#### ﻿ Purpurata lurida sp. nov.

Taxon classificationAnimaliaLepidopteraCrambidae

5E0B9CDA-5EBB-5422-A4E7-C3B6A82F2FC7

https://zoobank.org/04DDE7A6-D2E1-40AC-8D98-C6D9A526BB55

**Figures 17–20.**
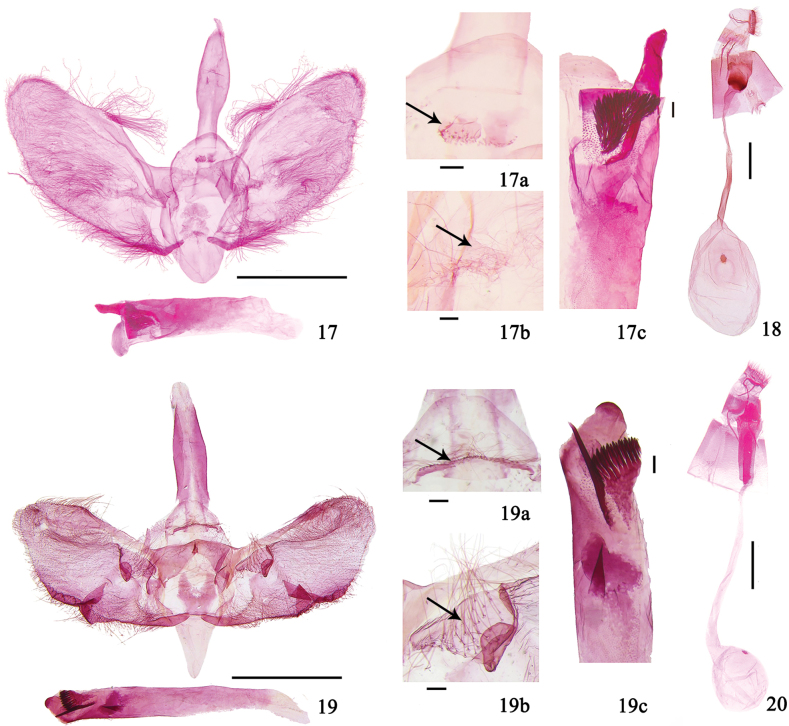
Genitalia of *Purpurata* species **17, 18***P.lurida* sp. nov. **17** male, holotype, genitalia slide no. XLJ14084 **18** female, paratype, genitalia slide no. LXQ18316 **19, 20***P.plagiatalis***19** male, genitalia slide no. HGQ13237 **20** female, genitalia slide no. XLJ14204 **17a, 19a** gnathos **17b, 19b** fibula **17c, 19c** cornuti and posterior protrusion of phallus. Scale bars: 1.0 mm (**17–20**); 0.1 mm (**17a–c, 19a–c**).

Figs 10 , 17 , 17a–c , 18

##### Diagnosis.

This species is similar to *P.obfuscalis* in appearance and genitalia, but can be distinguished by the body color paler than the latter (Fig. 10); uncus short and broad triangular (Fig. 17a); valva shorter and broader than the latter, with distal 1/3 narrowed very gradually and rounded apically (Fig. 17). In *P.obfuscalis*, the uncus is semicircular (Fig. 12a); the distal 1/3 of the valva is narrowed gradually and pointed apically (Fig. 12).

##### Type material.

***Holotype***: ♂ pinned, with genitalia in a separate slide. **China** • **Guizhou Prov.**, Kuankuoshui Nature Reserve, alt. 1500 m, 15 August 2010, Xi-Cui Du leg., genitalia slide number: XLJ14084. ***Paratypes***: pinned, some with genitalia in separate slides. **China** • **Hubei Prov.**, 2 ♂♂, Hejiaping Town, Changyang County, alt. 800 m, 18 June 2018, Xiao-Qiang Lu & Xi-Cui Du leg. • 2 ♂♂, Xingdou Mountain, Maoba Town, Enshi, alt. 780 m, 30 July 2012, Jun Zhang & Xiao-Bing Fu leg. • **Chongqing Municipality**, 2 ♂♂, Jinyun Mountain, alt. 550 m, 22, July 2010, Li Kang & Xing-Fu Fu leg., genitalia slide number: XLJ14103 • 2 ♂♂, 1 ♀, Jinyun Mountain, 29 July 2010, Xi-Cui Du & Chao-Wei Bi leg., genitalia slide number: XLJ14104 ♀ • 1♂, Jinyun Mountain, 30 July 2012, Li-Jun Xu & Jian-Bo Cao leg. • 2 ♂♂, Jinyin Mountain, Qianjiang District, alt. 1100 m, 26 July 2012, Li-Jun Xu & Jun-Zhang leg. • **Yunnan Prov.**, 1 ♂, Daxichang village, Malipo County, alt. 1465 m, 5 June 2015, Man-Fei Tao leg. • 2♀♀, Xishuangbanna Tropical Botanical Garden, alt. 659 m, 28 May 2015, Man-Fei Tao leg., genitalia slide number: LXQ18316 • **Hainan Prov.**, 1 ♂, Diaoluo Mountain vocational village, alt. 500 m, 23 May 2014, Li-Jun Xu & Dan Xu leg. • **Zhejiang Prov.**, 3 ♂♂, Tianmu Mountain, alt. 400 m, 24, 25, 30 July 2011, Xi-Cui Du & Xiao-Bing Fu leg.

##### Description.

***Habitus*** (Fig. 10). Forewing length 11.0–13.5 mm, wingspan 24.0–30.0 mm. Frons and vertex yellowish brown. Labial palpus with 1^st^ segment yellowish white ventrally, the remainder yellowish brown. Maxillary palpus brown. Antenna yellowish brown, with ventral cilia ~ 1/3 in length of diameter of flagellomere in male. Patagium and tegula yellow. Thorax yellow dorsally, white ventrally. Legs yellowish white, distal end of front tibia black. Wings pale yellow, with purple-brown lines and patches. Forewing with three small spots at base, another spot near basal dorsum; antemedial line slightly wavy, accompanied by a large elliptical pale patch inside; orbicular stigma a dark brown dot; discoidal stigma reniform, yellow centrally; postmedial line slightly obliquely inward from costa, dentate and excurved between M_2_ and CuA_2_, then incurved to discoidal stigma below and sinuous to inner margin; an irregular large patch near apex beyond anterior postmedial line, another misty patch near tornus below CuA_2_; a line of spots along marginal line. Hindwing with discoidal stigma a short oblique stripe; postmedial line same as forewing before CuA_2_, not apparent afterwards; an irregular large patch near apex beyond anterior postmedial line; a band below discoidal stigma, accompanied by another irregular misty wide band extended to tornus. Cilia of fore and hind wings yellowish white. Abdomen yellow dorsally, white ventrally; 1^st^ and 2^nd^ tergites with pale black spots laterally and 7^th^ tergites black posteriorly in male.

***Male genitalia*** (Fig. 17). Uncus short and broad triangular. Gnathos vestigial to a narrow band and bearing a few short setae (Fig. 17a). Valva broad tongue-shaped, with distal 1/3 narrowed very gradually, and rounded apically; costa arched medially and bearing a cluster of long setae; fibula a short lamina, setose basally (Fig. 17b). Saccus oval. Juxta a broad plate. Phallus posteriorly with a finger-like protruding sclerite, with a thick, needle-like cornutus and a brush-like cornutus composed of a spine cluster (Fig. 17c).

***Female genitalia*** (Fig. 18). Apophyses anteriores ~ 2 × length of apophyses posteriores. Antrum broad, ductus seminalis originating from antrum. Ductus bursae ~ 2 × length of corpus bursae. Corpus bursae nearly oval, with a round signum.

##### Distribution.

China (Chongqing, Guizhou, Yunnan, Hainan, Hubei, Zhejiang).

##### Etymology.

The species name *lurida* is derived from the Latin word *luridus*, an adjective, meaning pale yellow, indicating the pale wing color.

#### ﻿ Purpurata plagiatalis

Taxon classificationAnimaliaLepidopteraCrambidae

(Walker, 1859) comb. nov.

8EBAA078-F238-5540-BF37-1BD458999818

Figs 11 , 19 , 19a–c , 20

Botys plagiatalis Walker, 1859: 673. Type locality: Sri Lanka.
Pleuroptya plagiatalis : Inoue 1982: 1: 343, 2: 234.
Patania plagiatalis : Nuss et al. 2003–2024. Global Information System on Pyraloidea.

##### Material examined.

**Guangxi Zhuang Autonomous Region** • 1 ♂, Nonggang National Nature Reserve, Longzhou County, alt. 188 m, 27 July 2011, Gui-Qing He leg., genitalia slide number: HGQ13237 • 1 ♂, Mulun National Nature Reserve, alt. 288 m, 22 July 2015, Dan Xu leg. • **Hainan Prov.**, 1 ♀, Jianfengling Nature Reserve, alt. 770 m, 13 July 2014, Pei-Xin Cong, Lin-Jie Liu & Sha Hu leg. (NKU), genitalia slide number: XLJ14204.

##### Diagnosis.

This species is distinguished by wings with postmedial line punctiform and excurved between M_2_ and CuA_2_ (Fig. 11); gnathos vestigial to a narrow band (Fig. 19a); sacculus narrowed medially, with a lamellar projection near distal end (Fig. 19); fibula ear-shaped, with long setae (Fig. 19b); phallus posteriorly with a rounded protruding sclerite, with a thick needle-like cornutus and a brush-like cornutus, and a short thorn-like cornutus near posterior end (Fig. 19c).

##### Redescription.

***Habitus*** (Fig. 11). Forewing length 8.0–9.5 mm, wingspan 18.0–22.0 mm. Frons and vertex yellowish brown. Labial palpus yellowish white, pale brown at distal end of 2^nd^ segment. Maxillary palpus brown. Antenna yellowish brown, with ventral cilia ~ ½ in length of diameter of flagellomere in male. Patagium and tegula yellow, with pale yellowish brown patches centrally and basally respectively. Thorax yellow dorsally, white ventrally. Legs yellowish white, distal end of front tibia black. Wings yellow, with purple-brown lines and patches. Forewing with three small spots at base, another spot near basal dorsum; antemedial line slightly wavy, accompanied by a large elliptical patch inside; orbicular stigma a dark brown dot; discoidal stigma reniform, yellow centrally; postmedial line punctiform and excurved between M_2_ and CuA_2_; an irregular large patch near apex beyond anterior postmedial line, a nearly semicircular patch near tornus below CuA_2_; a line of small spots along terminal margin. Hindwing with discoidal stigma a short oblique stripe; postmedial line same as forewing before CuA_2_, not apparent afterwards; a large patch near apex beyond anterior postmedial line; a thin band below discoidal stigma, accompanied by an irregular misty wide band extended to tornus. Cilia of fore and hind wings yellowish white. Abdomen yellow dorsally, 1^st^ and 2^nd^ tergites with pale black spots laterally and 7^th^ tergites black posteriorly in male.

***Male genitalia*** (Fig. 19). Uncus short and broad. Gnathos vestigial to a narrow band and setose (Fig. 19a). Valva broad tongue-shaped with long setae along distal costa; fibula ear-shaped with long setae (Fig. 19b). Sacculus narrowed medially, with a lamellar projection near distal end. Saccus triangular. Juxta forcipate. Phallus posteriorly with a rounded protruding sclerite, with a thick needle-like cornutus and a brush-like cornutus composed of a spine cluster, and a short thorn-like cornutus near posterior end (Fig. 19c).

***Female genitalia*** (Fig. 20). Apophyses anteriores ~ 2 × as long as apophyses posteriores. Antrum relatively long, with a long, sclerotized band. Ductus bursae ~ 4 × as long as corpus bursae. Corpus bursae nearly rounded, with a round signum.

##### Distribution.

China (Xizang, Yunnan, Guangxi, Guangdong, Hainan, Fujian) (Lu and Guan 1953; Wang et al. 2003), Australia, Guatemala, Japan, India, Sri Lanka (Walker 1859; Inoue 1982; Klima 1939).

##### Host plants.

*Ipomoea* spp. (Wang et al. 2003).

#### ﻿ Purpurata shompen

Taxon classificationAnimaliaLepidopteraCrambidae

(Singh & Ahmad, 2022) comb. nov.

E4B98048-D516-566B-A014-B8652ABF5E82

Patania shompen Singh & Ahmad in Singh et al. 2022: 14, figs 1–2, 5–7. Type locality: India (Great Nicobar Island).

##### Diagnosis.

This species is similar to *P.iopasalis*, but can be distinguished by the postmedial line of forewing punctiform between M_2_ and CuA_2_, and the postmedial line of hindwing linear between M_2_ and CuA_2_ (Singh et al. 2022: fig. 1); phallus posteriorly with a rectangular protruding sclerite in male genitalia (Singh et al. 2022: fig. 7). In *P.iopasalis*, the postmedial line of the fore and hind wings are punctiform between M_2_ and CuA_2_ (Fig. 7); and phallus posteriorly with a nail head-like protruding sclerite in male genitalia (Fig. 14c).

##### Distribution.

India (Great Nicobar Island) (Singh et al. 2022).

##### Remarks.

This species is not found in China. The diagnosis is summarized based on the description and images of habitus and genitalia by Singh et al. (2022).

## ﻿Discussion

According to the description and habitus images by Moore (1888), we found the external morphology of *Syllepteleopardalis* (Moore, 1888), distributed in India, very similar to the new genus *Purpurata*, especially in wing color and markings. But its antennae do not seem to be filiform according to the habitus illustration provided by Moore. The genitalia of *S.leopardalis* were not described or illustrated, so further study is needed to confirm its morphological characters and affiliation.

## Supplementary Material

XML Treatment for Purpurata

XML Treatment for Purpurata obfuscalis

XML Treatment for Purpurata iopasalis

XML Treatment for Purpurata directa

XML Treatment for Purpurata lurida

XML Treatment for Purpurata plagiatalis

XML Treatment for Purpurata shompen

## ﻿Additional information

### Author ORCIDs

Xin-Lei Xue https://orcid.org/0009-0000-5697-0872

Xiao-Qiang Lu https://orcid.org/0009-0009-3696-7778

Xi-Cui Du https://orcid.org/0000-0002-7796-7303

### Data availability

All of the data that support the findings of this study are available in the main text.
